# Use of Functional Near-Infrared Spectroscopy to Predict and Measure Cochlear Implant Outcomes: A Scoping Review

**DOI:** 10.3390/brainsci11111439

**Published:** 2021-10-28

**Authors:** Samantha C. Harrison, Rachael Lawrence, Derek J. Hoare, Ian M. Wiggins, Douglas E. H. Hartley

**Affiliations:** 1 NIHR Nottingham Biomedical Research Centre, Nottingham NG1 5DU, UK; rachael.lawrence@nottingham.ac.uk (R.L.); Derek.Hoare@nottingham.ac.uk (D.J.H.); ian.wiggins@nottingham.ac.uk (I.M.W.); douglas.hartley@nottingham.ac.uk (D.E.H.H.); 2 Hearing Sciences, Mental Health and Clinical Neurosciences, School of Medicine, University of Nottingham, Nottingham NG1 5DU, UK; 3 Nottingham University Hospitals National Health Service Trust, Nottingham NG5 1PB, UK

**Keywords:** fNIRS, speech perception, plasticity, objective measures

## Abstract

Outcomes following cochlear implantation vary widely for both adults and children, and behavioral tests are currently relied upon to assess this. However, these behavioral tests rely on subjective judgements that can be unreliable, particularly for infants and young children. The addition of an objective test of outcome following cochlear implantation is therefore desirable. The aim of this scoping review was to comprehensively catalogue the evidence for the potential of functional near infrared spectroscopy (fNIRS) to be used as a tool to objectively predict and measure cochlear implant outcomes. A scoping review of the literature was conducted following the PRISMA extension for scoping review framework. Searches were conducted in the MEDLINE, EMBASE, PubMed, CINAHL, SCOPUS, and Web of Science electronic databases, with a hand search conducted in Google Scholar. Key terms relating to near infrared spectroscopy and cochlear implants were used to identify relevant publications. Eight records met the criteria for inclusion. Seven records reported on adult populations, with five records only including post-lingually deaf individuals and two including both pre- and post-lingually deaf individuals. Studies were either longitudinal or cross-sectional, and all studies compared fNIRS measurements with receptive speech outcomes. This review identified and collated key work in this field. The homogeneity of the populations studied so far identifies key gaps for future research, including the use of fNIRS in infants. By mapping the literature on this important topic, this review contributes knowledge towards the improvement of outcomes following cochlear implantation.

## 1. Introduction

Hearing loss is believed to be the most common cause of moderate to severe disability worldwide [1], and it is estimated that 1 in 10 people worldwide will be living with disabling hearing loss by 2050 [2]. Hearing loss can be present at birth or can develop later in life. Severe congenital hearing loss may lead to psychological, educational, and linguistic deficits if left unchecked [3], and many countries have therefore implemented newborn hearing screening programs for early identification and hearing intervention [4]. Mild hearing loss and acquired deafness can also be detrimental to an individual’s health-related quality of life [5,6] as well as to their educational and/or vocational abilities [7].

In cases of severe to profound hearing loss, individuals can receive cochlear implants (CIs) to partially restore hearing ability. CIs are neuro-prosthetic devices that convert acoustic signals from the environment into electrical signals that directly stimulate the auditory nerve via an array of electrodes that are surgically implanted in the inner ear. The benefits of CIs include improved communication skills and faster language acquisition in children [8], awareness of environmental sounds [9], and in the case of bilateral implantation, improved speech localization [10]. It is possibly because of these improvements that CI users also report improved general quality of life [11,12,13].

Despite the benefits of Cis, there remains large individual variability in the outcomes between CI recipients, both in pre-lingually [14,15,16] and post-lingually deafened [17,18,19] populations. Even when controlling for factors such as the duration of profound hearing loss, etiology, duration of hearing aid use, and duration of CI experience, over 75% of the variance in CI outcomes remains unaccounted for [19,20,21].

Variable outcomes can be particularly problematic in the case of infants receiving CIs [22]. This is because there is no method to objectively measure outcomes until some months or years after the procedure, and there is a lack of reliable behavioral measures of speech perception for infants and young children. This means that individuals struggling with their implants may not receive any additional interventions and support until later in development.

It has long been accepted that sensory deprivation causes anatomical and functional changes in animal [23,24,25] and human brains [26]. Modality-specific areas of the brain can be utilized for processing information from a different modality, i.e., cross-modal plasticity (also referred to as cross-modal takeover and cross-modal reorganization). In blind individuals, there is evidence of auditory-evoked and tactile-evoked activation in the visual cortex [27,28]. In the case of auditory deprivation, auditory regions of the brain have been evidenced to respond to non-auditory stimulation such as vibrotactile and visual information in both post-lingually [29] and pre-lingually deaf populations [30,31,32,33,34,35,36]. Additionally, the recruitment of visual areas to process speech stimuli has also been shown in post-lingually deaf cochlear implant users [37,38], thus demonstrating that the cross-modal plasticity caused by auditory deprivation is not limited to the auditory cortical regions.

In addition to cross-modal plasticity, further intra-modal plasticity has been evidenced in pre-lingually deaf individuals. Whereas cross-modal plasticity refers to modality-specific areas of the brain being utilized to process stimuli from a different modality, intra-modal plasticity refers to changes within modality-specific areas of the brain as they process stimuli from that specific modality. Research using electroencephalography (EEG) revealed a reduction of visually evoked responses in the visual cortical system of deaf adults compared to hearing controls [35]. Furthermore, studies have shown faster neural processing of visual stimuli in the visual regions of deaf adults compared to hearing controls [39]. These results suggest that there are changes to visual processing within the visual cortex due to the impact of auditory deprivation.

This functional reorganization could be viewed as adaptive; in studies of blind individuals, increased tactile discrimination abilities compared to sighted controls have been suggested to be in parallel with cross modal-plasticity [40]. Enhanced abilities of non-visual skills such as sound localization and verbal memory have been evidenced to be positively related to levels of cross-modal plasticity [41,42]. Further, studies using transcranial magnetic stimulation (TMS) demonstrate impaired Braille reading performance and verb generation when the visual cortex is disrupted in blind individuals [43,44], suggesting that these enhanced abilities are related to cross-modal plasticity. In studies of pre-lingually deaf individuals, neural plasticity is in parallel with enhanced abilities of non-auditory skills such as tactile sensitivity [45], motion detection and discrimination [46,47], and peripheral field and attention [48,49]. This supports the notion of adaptive cross-modal plasticity after auditory deprivation.

Contrastingly, there are instances where cross-modal plasticity has been believed to be maladaptive; this is primarily in cases of sensory restoration. Cochlear implantation uniquely allows for the exploration of what happens when a previously deprived sensory modality is restored. Here, concerns have been raised regarding how cross-modal plasticity may hinder the ability of auditory cortical regions to perform their primary function post-implantation [50]. Research exploring CI outcomes has revealed that duration of deafness is an important factor in CI success [19,20], and this has been attributed to increased levels of cross-modal plasticity hindering the auditory cortical regions from processing the newly introduced auditory stimuli. Similar findings have been demonstrated in positron emission tomography (PET) [51,52,53] and visual-evoked potential (VEP) [50,54,55] studies in which evidence of cross-modal plasticity is linked to poorer speech performance outcomes in CI users.

However, it has been argued that this adaptive versus maladaptive stance is overly simplistic [56]. Instead, the activation of auditory cortical regions by visual linguistic information may not limit the recovery of the auditory sense post-implantation but rather can aid in the preservation of key language networks, which, in turn, may help improve CI outcomes [57,58].

A wide body of neuroimaging literature explores these arguments further by exploring the relationship between cortical activation and cochlear implant outcomes. For example, research using fMRI on post-lingually deaf adults has demonstrated a negative correlation between pre-surgical cortical activation of the right supramarginal gyrus, an auditory region typically involved in processing non-linguistic speech information such as pitch, during a phonological judgement task and speech perception scores measured post-implantation [59]. In infants, machine learning algorithms have been shown to successfully use neuroanatomical information from pre-implantation MRI scans to predict post-implantation success in CI users aged 8–38 months [60]. This has been attributed to increased levels of cross-modal plasticity impacting how the brain processes newly introduced auditory stimuli from the CI.

A clinically suitable way of further understanding and monitoring how patterns of cortical activity such as cross-modal plasticity relate to variability in CI outcomes could help inform prognoses. Evidence of cross-modal plasticity pre-operatively could be used to predict the likelihood of success, and post-operative responses to auditory stimuli and evidence of cross-modal plasticity could be used to monitor subsequent adaptation to the restored auditory input. Access to such objective evidence would be useful within clinical settings and would support adequate and timely rehabilitation and support interventions being put in place.

However, the neuroimaging of CI recipients, particularly when repeated before and after surgery, has been notoriously difficult because of the limitations of traditional imaging methods. For example, using functional magnetic resonance imaging (fMRI) for auditory research can be problematic because of the impact of extraneous scanner noise [61,62]. Additionally, fMRI is particularly susceptible to movement artefacts, meaning infants and young children often must be sedated or asleep during scanning. Furthermore, the imaging of the temporal areas of the brain using MRI methodologies is problematic post-implantation because of the artefacts associated with the CI magnet. Magnetic artefacts are also problematic for magnetoencephalography (MEG). EEG data can be limited by implant-related electrical artefacts—though new analysis techniques are being developed to overcome this [63,64]. Some studies have successfully used PET to image the brain following cochlear implantation [65,66,67]. However, this technique requires the injection of radioactive isotopes, making it unsuitable for repeated use or for use in infants and children because of the cumulative effects of radionucleotide exposure. Optical neuroimaging techniques such as functional near infrared spectroscopy (fNIRS) do not have these limitations.

fNIRS is increasingly used for functional brain-imaging research in adults and children, including CI recipients, because it is quiet, non-invasive, and CI-compatible [68,69,70,71,72,73,74,75]. fNIRS uses near infrared light and as such is unaffected by electrical or magnetic artefacts. This means that fNIRS imaging can be conducted across most of the outer cortex and is only restricted in the regions directly on top of the CI transmitter/receiver sites, where the near infrared light cannot penetrate.

fNIRS has been used to explore cross-modal plasticity in adults with hearing loss [76]. There have also been several fNIRS studies that have been conducted involving adults and children with CIs [77,78,79,80]. This work with CI users demonstrated levels of cross-modal plasticity [78,80], overlap in the processing of the features of auditory speech between CI users and normally hearing children [78], and an overall neurotypical pattern of activation during auditory language tasks [77]. Crucially, all of this work has demonstrated the utility and tolerability of this technique for studies with CI-users. Because fNIRS is non-invasive, relatively inexpensive, and portable, this imaging method could be useful clinically as an objective measure or predictor of CI outcomes.

There have been a number of reviews regarding the use of fNIRS in auditory/language research [70,81], and more specifically, the use of fNIRS in deaf or CI-using populations [73,82]. However, to the best of our knowledge, no literature is available that specifically reviews work assessing the relationship between fNIRS cortical measures and behavioral outcomes in CI users. The aim of the review was to determine what has already been done in the field and where the opportunities and gaps to be addressed in the future are. Therefore, the objective was to catalogue research that has used fNIRS imaging to measure or to predict CI outcomes, which outcome measures have been used, and which populations have been studied.

## 2. Materials and Methods

This review employed a scoping review methodology [83] and is reported according to the PRISMA extension for scoping reviews (PRISMA ScR) [84,85].

### 2.1. Eligibility Criteria

A two-stage screening process was used to assess the relevance of the records identified from the searches. Records were eligible for inclusion if they were peer-reviewed reports on research with CI recipients and compared results from a NIRS-based methodology to a measure of CI outcome. No limits were placed on the searches with regard to publication language or date to allow for an unhindered exploration of the field.

### 2.2. Information Sources

The electronic databases MEDLINE, EMBASE, PubMed, CINAHL, SCOPUS, and Web of Science were searched to identify peer-reviewed literature. Google Scholar and the reference lists of included records were searched to identify other literature not captured in the database search.

### 2.3. Searches

Key concepts and search terms were established to identify literature related to the fNIRS imaging of CI users. Techniques for our search included the use of Boolean operators to narrow, widen, and combine searches, depending on the database. An example of the full search strategy in PubMed is included in Supplementary Digital Content 1.

All database searches were conducted in June 2020. A hand search of Google Scholar was also conducted by SH in June 2020, with a stopping rule of two successive pages of results with no new records identified for inclusion. Additionally, a hand search of the reference lists and citation lists of included articles was undertaken across June–July 2020. A final update search of Google Scholar was conducted in February 2021 (limited to 2020–2021) to identify any further records that had been published since June 2020.

### 2.4. Selection of Sources of Evidence

Search results were imported into an online systematic review software (Covidence systematic review software n.d.). Eligibility criteria were imported and were used to screen the titles and abstracts. All eligible records proceeded to full-text screening, where the eligibility criteria were applied again. Both screening stages were completed by SH and RL independently. Any discrepancies between reviewers were discussed, and agreements were reached without the need for an arbitrator.

### 2.5. Data Charting Process

A data chart was developed in Excel and was piloted by SH and DJH. Data extraction was completed by SH. RL confirmed the accuracy of all of the information within the chart.

### 2.6. Data Items and Synthesis of Results

For all of the included articles, summaries were developed by outlining key information including publication year, main purpose/research questions, sample population and size, stimuli used, cortical regions of interest, fNIRS details, outcomes and measurements, study design, and main results. Nominal data were described with frequencies.

## 3. Results

### 3.1. Selection of Sources of Evidence

**Figure 1:**
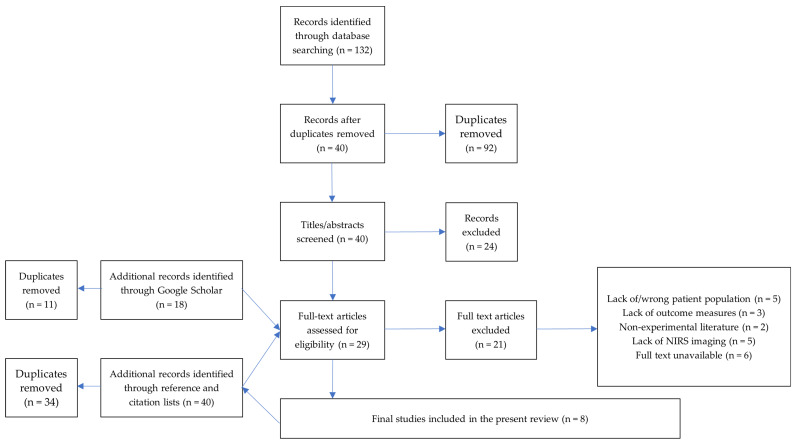
Flowchart to represent the search and screening process. A total of eight articles were deemed appropriate for this review.

Figure 1 illustrates the record selection process used for this review. Searches generated across all databases excluding Google Scholar yielded 132 articles, of which 92 were immediately removed as duplicates. The title and abstract of the remaining 40 records were screened, with 24 articles excluded as not meeting all criteria. The remaining 16 records were subjected to full-text screening. Ten were excluded, leaving six to be included from these searches.

The initial Google Scholar search generated 16,300 records, of which 11 pages (110 results) were screened before the stopping rule applied. Twelve potentially eligible records were identified, and ten duplicates were removed. Two articles progressed to full-text screening, resulting in one record being excluded and the other being included. Thus, seven records were eligible for inclusion at that stage. An additional 40 records were identified from the reference and citation lists of these included records. After 34 duplicates were removed, 6 articles were subject to full-text screening, and all 6 were excluded.

The final update search generated 341 records, of which 6 pages (60 results) were screened before the stopping rule applied. Six potentially eligible records were identified and after a duplicate was removed, five progressed to full text screening, where four records were excluded. Thus, eight records met all of the criteria for inclusion in this review.

### 3.2. Characteristics of Sources of Evidence

Three records reported studies from the UK [78,86,87], three reported studies from Germany [88,89,90], one reported a study from the US [91], and one study was from Australia [92]. All of the included studies were published between 2016 and 2020. As no date limits were imposed during the initial literature searches, this demonstrates the novelty of the field.

### 3.3. Results of Individual Sources of Evidence

Summaries of the sampling and design information of the included articles are given in Table 1. Table 2 identifies the research questions and key results. It is worth noting here that some of the articles included identical samples and exclusion criteria. Confirmation was found within these articles that they were based on the same original research study. Thus, it was concluded that only five separate studies were conducted in this area that resulted in the eight identified records.

### 3.4. Synthesis of Results

Out of the eight included records, seven focused solely on adult participants. The remaining article included child participants who were 6-years-old or older. Whilst five articles included only post-lingually deaf participants [88,89,90,91,92], two included a sample with both pre- and post-lingually deaf participants [86,87], and one article included a sample with only pre-lingually deaf participants [78]. Two articles followed participants from pre- to post-implantation [86,87]. The other six articles were all conducted post-implantation but varied in length of participant CI experience [78,88,89,90,91,92]. Three articles studied CI users with at least 6 months post-implantation experience [88,89,90], one article defined CI experience as more than 12 months [92], one article noted that the shortest length of CI experience in their sample was 29 months [78], and contrastingly, one article included participants with a range of experience from 1 day to 12 years [91].

All eight articles included only healthy participants, with examples of exclusion criteria including anyone with a history of “language, cognitive or motor disorder or brain injury” [86] and anyone with a “history of neurological or psychiatric illness” [88,89,90].

Only two records were longitudinal, meaning that they examined fNIRS as a predictor of CI outcomes [86,87]. The other six articles reported cross-sectional studies and thus examined fNIRS as a measure of CI outcomes [78,88,89,90,91,92]. All of the included records examined speech perception by using behavioral measures such as CUNY sentence lists (City University of New York) [93] in quiet or the Oldenburg sentences test (OLSA) [94].

## 4. Discussion

The records included in this review stem from four laboratories around the world. This shows that the current capacity for work into the use of fNIRS as a measure or predictor of CI outcomes is very low and that significant time and investment is needed to propel the field forwards. Nevertheless, it is important to summarize the work so far so that a clear path can be established for future research.

### 4.1. Overview of Results from Research in This Field

Some articles within this review explored visually evoked activation in the auditory cortical regions of adults [86,87,92] and children [78]. Stronger visually evoked activation of the auditory cortical regions was negatively correlated with speech understanding outcomes, both when measured post-implantation [92] and when measured pre-implantation and compared to post-implantation speech understanding [87]. This could suggest that visual takeover of the auditory regions during deafness is maladaptive to CI outcomes, potentially inhibiting the auditory cortical regions from adequately processing auditory stimuli. However, Anderson et al. noted no association between pre-implantation visual processing and post-implantation responsiveness to auditory speech [86], suggesting that this maladaptive view may not be as simple. Instead, their longitudinal work found that an increase in the visual activation of the auditory regions post-implantation was positively correlated with speech understanding outcomes, suggesting instead that visual processing in the auditory regions can aid in post-implantation speech processing [86]. It should be noted that the visual stimuli used was visual speech (i.e., lipreading). This could suggest that CI users utilize visual speech information to help them understand their new auditory stimulation, and therefore an increase in visual processing in the auditory regions after implantation could be adaptive. Contrastingly, in a study of children with CIs, no relationship was identified between the visual activation of the auditory regions and CI outcomes [78].

Another area of interest was that of responses in the auditory cortical regions to intelligible versus unintelligible speech. The results showed no correlation between intelligibility processing and CI outcomes in children [78]. In adults, the results demonstrated that CI users with good outcomes had stronger cortical responses to intelligible speech versus scrambled speech, whereas CI users with poorer outcomes had no distinguishable differences in the processing of the two stimulus types [91]. This suggests that, at least in adults, CI outcomes rely on the brain’s ability to differentiate between intelligible speech and other auditory stimulation.

As well as cross-modal activation, cross-modal functional connectivity (a statistical relationship between activity in two or more distinct brain regions) between visual and auditory cortical regions correlated negatively with speech understanding scores measured by the Freiburg monosyllabic words test [88]. Interestingly, a significant correlation was not found when speech understanding was measured by the OLSA test. Speech understanding measured by the OLSA test also did not correlate with levels of adaptation to auditory stimuli, which are categorized by a decrease in activation to a repeated stimulus [90]. However, speech understanding measured by the OLSA test was positively correlated with the ratio by which cortical reorganization was seen in the visual versus auditory cortical regions [89].

As can be seen here, this is a promising start to the field, though a lack of cohesion across studies conducted so far makes it difficult to draw any solid conclusions or make comparisons of the results. Instead, at this stage, it is important to discuss the methodological aspects of the research that has been conducted. This will enable the field to identify key opportunities and gaps to be addressed in the future.

### 4.2. Populations That Have Participated in Research in This Field

In terms of research exploring the use of fNIRS as a measure/predictor of CI outcomes, most of the work is in adult populations, and work has begun with samples of children aged 6 years and older [78]. There has, however, been no published work in infant populations. This gap in the literature is important to highlight because infants arguably have the most to gain from a clinical tool that measures or predicts CI outcome. As infants are believed to pass various sensitive periods of language acquisition during early development [95], it is crucial that optimal rehabilitation is offered as early as possible in order to give the best chance of more effective results. The use of an objective tool such as fNIRS, which can be used with all ages, would mean that pediatric patients with potentially poor outcomes can be identified earlier and thus could receive earlier, more tailored interventions.

This review also revealed that so far, the work in this field has mostly been conducted on post-lingually deafened populations. It is well established that prior hearing experience is associated with good CI outcomes [19,20,21], and this can be attributed to several factors such as age at deafness onset and hearing aid usage. In line with this, there are marked differences in the association between fNIRS responses and behavioral results between pre- and post-lingually deafened populations [87]. Namely, there was a trend of adaptive cross-modal plasticity, where visually evoked activation of the auditory regions was positively correlated with CI outcomes in a pre-lingually deaf sample but not in the post-lingually deaf sample [87]. Given the strong levels of heterogeneity within the cochlear implanted population, this means that the interpretation of the fNIRS results for the purpose of measuring or predicting CI outcomes will need to be carefully considered alongside other clinical factors, such as onset of deafness, if an fNIRS-based tool is trialed in clinics. Importantly, the inconsistency in the adaptive qualities of cross-modal plasticity, as reported by Anderson et al. [87], also demonstrates the need for more research exploring the mechanisms behind the relationships between plasticity, clinical factors, and outcome.

All of the records included in this review recruited CI-users who were otherwise healthy, excluding participants at the recruitment stage for reasons such as cognitive disorder, neurological illness, or brain injury. However, it is well documented that individuals with hearing loss often have other comorbid conditions, such as developmental delay, autism spectrum disorder or cerebral palsy in children [96,97], and cognitive and psychological impairments in adults [98,99]. Whilst the inclusion or exclusion of individuals with such conditions from research studies is justifiable during early work in this field, it will be important to consider at what stage future work will include participants on such grounds and how these decisions might impact the applicability of a future clinical tool.

### 4.3. Clinical Outcomes the Field Has Tried to Measure or Predict with fNIRS Imaging

All of the studies that were catalogued in this review used speech perception abilities as an outcome measure. Given that the key aim of cochlear implantation, particularly for infants and young children, is to maximize the ability to perceive auditory speech, it is appropriate to use this as an outcome measure. However, the method used to measure speech perception abilities was not consistent across studies. Some tests involved listening to auditory speech in quiet, whilst others involved listening to speech in noise. Additionally, as noted in one record, some participants performed near ceiling on the speech perception task [87], and so task sensitivity needs to be carefully considered to allow for the accurate measurement of speech perception across the range of participants. One way to do this could be to assess the speech recognition threshold. This examines the level at which speech stimuli can be correctly repeated 50% of the time and thus avoids the ceiling effects seen in other assessments. As this field develops, it may be useful to agree upon a core set of CI outcomes that are important to patients and associated tests so that more direct comparisons can be made between studies conducted by different research groups. Consideration needs to be given to the challenges of conducting research in languages other than English and to comparisons of results derived from populations who speak different languages. Agreeing upon a core set of outcomes and associated tests that can be standardized and validated across multiple languages will allow for the quicker identification of key research outcomes and greater data synthesis.

### 4.4. fNIRS Measurements

All of the records included in this review reported bilateral fNIRS imaging and focused on the region of the temporal lobe, with some specifying the superior temporal cortex (STC) as the cortical region of interest. Some studies examined the degree of STC activation and, more specifically, the degree of cross-modal activation by visual speech and how predictive this is of speech perception outcomes post-implantation [86,87]. Others explored the degree of intra-modal and cross-modal functional connectivity within and between the visual and auditory areas [88,89,90]. All adult studies found significant relationships between their employed fNIRS measures and speech perception outcome measures, but this was not the case for child participants [78] (see Table 2 for the results from each study). This difference may be due to the inclusion of only pre-lingually deaf participants or due to the ongoing cortical development in pediatric populations. More work with pediatric samples is needed to further explore the impact that pre-lingual deafness and early cortical development have on the relationships between fNIRS results and CI outcomes.

It is important to note that one record included in this review found that the relationship between fNIRS measurements and outcomes is driven by additional factors, i.e., whether the participants are pre- or post-lingually deaf [87]. Hence, the specific details of the fNIRS measurement and subsequent comparisons to behavioral outcomes should be determined by the qualities of the clinical population being tested.

### 4.5. Implications for Future Research

As this review reveals, the field of utilizing fNIRS as an objective measure of CI outcomes is relatively young. The use of fNIRS as a tool for measuring or predicting CI outcomes has only appeared in the scientific literature since 2016, and as such, there are many directions that future work could take.

#### 4.5.1. Heterogenous Samples

Significant heterogeneity exists within the CI user population. This heterogeneity may include differences in age, age at onset of deafness, hearing aid usage, residual hearing levels, and surgical techniques used, to name just a few. These factors have a clear impact on CI outcomes. For example, a late onset of deafness is associated with better CI outcomes, as are higher levels of hearing aid usage and greater residual hearing [20]. Soft-surgery techniques, which seek to preserve residual hearing, are also associated with better CI outcomes [100]. The relationship between these factors and cortical activation is an area of active investigation, but it is possible that many of these factors have a distinct impact on levels of cross-modal plasticity (and thus activation recorded with fNIRS), so it is crucial that research in the field considers samples with a range of ages, backgrounds, experiences, and etiologies. This includes sampling both pre- and post-lingually deaf populations whilst noting the possibility that relationships between cortical functioning and behavioral outcomes may be qualitatively different between these two sub-populations.

#### 4.5.2. Pediatric and Geriatric Research

When considering sampling, it is crucial to consider the age of the population. The only pediatric study identified in this review included a sample of children aged six years and older. However, it is important to also begin work with infant populations. The use of an objective tool that is appropriate for all ages, such as fNIRS, would mean that infants who are at a predictable risk of poorer outcomes could be identified much earlier and thus could receive earlier interventions before sensitive periods of language acquisition have passed. This, in turn, could promote better speech perception, improving educational and vocational achievements, social interactions, and quality of life [9,101,102].

fNIRS has previously been used to examine cortical responses in paediatric CI users. For example, early fNIRS work in paediatric CI users determined the utility of fNIRS for studying auditory cortical responses, both at switch on and after at least 4 months of CI use in children aged 2 years and older [79]. However, most of these paediatric studies did not explore the relationships between fNIRS measurements and CI outcomes; thus, only one was included in the present review [78], and this study only included children above 6 years of age.

It must be noted that fNIRS imaging of infants and young children brings a new set of challenges not typically seen in studies of older children and adults. For example, higher data contamination by movement artefacts is evident in fNIRS research with awake infant participants. Rejection of contaminated trials is undesirable due to the constraints on the amount of data collected due to limited infant tolerance and attention span. Therefore, motion correction is preferable to reduce the number of trials that need to be rejected from analysis [103,104], so careful consideration should be given to the data pre-processing stages of fNIRS work with awake infant participants.

Furthermore, differing hemodynamic responses have been identified using both fNIRS and fMRI between adult and infant studies. Specifically, whereas adult hemodynamic responses are typically canonical (an increase in oxy-hemoglobin and a simultaneous decrease in deoxy-hemoglobin), infant hemodynamic responses have been seen to vary from canonical to inverted (an increase in deoxy-hemoglobin and a simultaneous decrease in oxy-hemoglobin) or to significant changes to both chromophores in the same direction [105,106,107]. This may be due to physiological differences between the populations, with standard neurovascular coupling still developing in young infants [108]. Alternatively, the observed variation may be due to inter-study differences, such as the waking state of the infant or the paradigm employed [109,110]. Regardless, the topics discussed in this section demonstrate the importance of conducting fNIRS research on infants and young children, rather than relying on results from studies of adults and older children, if an objective tool is to be used with paediatric patient populations. As well as work with infant populations, future work in this field may also want to consider exploring differences between younger and older adult populations. Research has demonstrated poorer CI outcomes in older adult populations compared to their younger counterparts, particularly with regard to perceiving speech in noise [111,112,113,114]. This mirrors work in non-CI populations, which demonstrates lower levels of speech in noise perception abilities in older compared to younger adults [115]. In part, this may be due to the peripheral hearing loss often seen in older populations [116]. However, there may also be a role of cognitive processes, such as attention and phonological working memory, which are more heavily relied upon for speech perception in older adults. For example, research has demonstrated positive correlations between cortical activity in cognitive areas and speech perception in noise abilities in older adults without CIs but not in younger adults [117]. Similarly, a positive correlation between the thickness of the cognitive cortical structures and speech perception in noise abilities in older adults without CIs has been found [118], strengthening the argument that cognitive cortical regions are important for speech perception outcomes in this population and thus may play a part in CI success in older adults.

As the research identified in this review focused on sensory cortical regions in samples of adults across broad age ranges, further exploration of cognitive cortical activity may be useful, particularly alongside an assessment of age-related differences in the relationship between cortical activity and CI outcomes in the adult population. It is plausible that an objective tool may need to be used to identify different patterns of cortical activity for younger and older adult patients so that it is able to accurately predict, measure, or monitor CI outcomes across the age ranges.

#### 4.5.3. Outcome Measures

In all of the research mapped thus far, the ability to perceive speech has been used as the primary measure of CI outcomes. However, additional related abilities may be useful to test the specificity and versatility of an fNIRS-based measure or predictor of CI outcomes. Expressive language (the ability to produce language) and higher-level language skills are key to educational, professional, and social proficiency. fNIRS has been used in the clinical research setting, for both pediatric and adult patients, as a presurgical assessment of expressive and receptive language, cortical lateralization, and subsequent surgical candidacy for refractory epilepsy [119,120]. Hence, the use of fNIRS to investigate and predict expressive and higher order language functions and outcomes in the CI population is a potentially exciting prospect.

Further insight might be gained by using fNIRS to study brain responses to more basic speech tokens, or non-speech auditory stimuli. For example, previous fNIRS studies have explored cortical responses to phonemes [121,122], sound intensity [123], prosody [124,125,126,127], and music [128,129]. Stimuli such as these are removed from the contextual and cognitive effects of perceiving full words and sentences and therefore present an interesting direction to extend the current literature on the relationship between fNIRS measurements and CI outcomes.

Additionally, observations suggest that there may be a relationship between executive functions, top-down cognitive processes utilised to achieve goal-directed actions, and language, as they both emerge and develop during childhood [130]. In line with this, published research suggests pre-lingually deafened children with Cis are two to five times more likely to have delayed executive functioning behaviours compared to children with normal hearing [131]. Therefore, it may be useful to explore the relationship between executive functioning and CI outcomes, how this relationship is presented on a cortical level, and whether this cortical presentation is suitable as an objective measure and/or predictor of CI outcome.

Most of the research discussed in this review was cross-sectional, meaning that the fNIRS and behavioral measurements were collected once per participant. Cross-sectional work is important here because an objective tool could be used to measure and monitor a CI recipient’s progress as well as support CI programming and calibration. Alternatively, an objective tool could be used to predict longer-term outcomes and support expectation management and personalized care planning and implementation. Additionally, this could be useful for patients where regular post-implantation appointments are less common because of distance or funding restrictions; a measurement tool only gives here-and-now information, but a predictive tool could cover longer term assessment. It is important that future work considers a longitudinal study design where participants are tested over multiple time periods to explore changes, which will allow for both measurement and prediction viability to be assessed.

#### 4.5.4. Imaging Techniques

Although not specifically discussed in the studies identified in this scoping review, achieving a more robust estimation of fNIRS measurements is vital to be able to use fNIRS as a clinical tool on a patient-by-patient basis. fNIRS has demonstrated good to excellent test–retest reliability for measuring cortical responses in the temporal cortices at a group level, though high levels of variability were evident among individuals [132,133]. Similar results have also been demonstrated in the frontal [134,135], sensorimotor [136], and visual [137] cortical regions. Whilst the temporal resolution of fNIRS is greater than that of fMRI, for example, it is less than that of EEG. Conversely, the spatial resolution of fNIRS is greater than EEG but less than that of fMRI. Whilst this makes fNIRS a good compromise for many research purposes, greater accuracy and greater reliability at a single-subject level is needed to apply this technique to medical settings. This may be feasible through use of high-density fNIRS systems and/or the combination of fNIRS with other CI-conditional imaging techniques such as EEG. Research has shown that the use of high-density fNIRS imaging using multi-distance optode separations contributed significantly to the accuracy of an fNIRS brain–computer interface [138]. In terms of simultaneous fNIRS and EEG imaging, this system offers improved spatial and temporal resolutions in addition to whole brain measurement [139]. Combined fNIRS and EEG imaging have been used in combination to successfully examine and increase the classification accuracy of visual and auditory stimulus processing [140,141]. This application could feasibly be applied to CI recipients to improve the accuracy of the measurements and prediction of CI outcomes using cortical measures.

### 4.6. Limitations

As this review was primarily interested in what studies of fNIRS have been published, it only used searches of academic databases and reference lists of relevant peer-reviewed records. From this, only peer-reviewed studies were included. Additional relevant studies may have been identified if grey literature records such as theses or preprints or general search engines were used.

## 5. Conclusions

This scoping review mapped research conducted to date on the use of fNIRS to measure or predict CI outcomes. Here, we specifically reviewed work assessing the correlation between fNIRS cortical measures and behavioral outcomes in CI users. This field is young, with work only published in the last 5 years. Over this time period, promising initial results have been obtained that suggest that it may be possible to develop an objective fNIRS-based clinical tool. However, much work remains to be completed before such a tool would be ready for clinical application. Work so far has primarily focused on adult CI users, most of whom were post-lingually deafened. However, with extensive heterogeneity in the CI-using population, future research needs to consider more varied population samples, both in terms of age and medical history. To date, published work has been consistent in terms of the outcomes that have been explored but has also employed varied methods of quantifying these outcomes. Overall, this field has made good progress, but more work needs to be conducted before the promise of an objective fNIRS-based tool can be realized. An important next step is for the field agree on a core set of CI outcomes and outcome measures. Consideration should be given to measures that seek to quantify thus far unexplored outcomes such as expressive language and executive function.

## Figures and Tables

**Table 1:** Sampling and design information from the eight included articles.

Record	Sample	Stimuli/Imaging Paradigm	Cortical ROIs	Outcome & Measurements	Study Design
Anderson et al., 2017 [86]	Patient group: 17. Bilaterally profoundly deaf, pre-surgical. Two pre-lingually, three peri-lingually, and twelve post-lingually deaf. Age 36–78 (mean = 58). Controls: 17. Mean age = 57 years.	IHR number sentences (normal speech, male and female speakers). Split into visual-only, auditory-only. All at 65 dB for 24 s blocks	Bilateral fNIRS with lowermost optode close to preauricular point and uppermost optode aligned towards Cz. Targets temporal lobe, specifically superior temporal cortex (STC)	Speech understanding: CUNY (City University of New York) Sentence lists in quiet. Measured via speech reading pre-implantation and via auditory performance post-implantation.	Longitudinal repeated measures
Anderson et al., 2019 [87]	Patient group: 17. Bilaterally profoundly deaf, pre-surgical. Mix of pre- and post-lingually deaf. Age 36–78 (mean = 58). Controls: 17. Mean age = 57 years.	IHR number sentences (normal speech, male and female speakers). Split into visual-only, auditory-only, audio-visual. All at 65 dB for 24 s blocks	Bilateral fNIRS with lowermost optode close to preauricular point and uppermost optode aligned towards Cz. Targets temporal lobe, specifically superior temporal cortex (STC)	Speech understanding: CUNY (City University of New York) Sentence lists in quiet. Measured via speech reading pre-implantation, and via auditory performance post-implantation.	Longitudinal repeated measures
Chen et al., 2017 [88]	Patient group: 20. Unilaterally implanted post-lingually deaf CI users with ≥6 months experience. Age 24–77 (mean = 54.58). Controls: 20. Age 24–78 (mean = 54.89).	Visual stimuli consisting of circular checkerboard patterns in 10 s blocks. Auditory stimuli consisting of normal speech and reversed speech in 5 s blocks and tonal bursts in 3 s blocks. Loudness levels for auditory stimuli were adjusted to subjective comfortable levels.	Bilateral fNIRS. Temporal lobe headset centered at T7/T8. Occipital lobe headset centered at O1/O2.	Speech recognition: Freiburg monosyllabic words test, Oldenburg sentences test (OLSA) in quiet, OLSA test in noise.	Cross-sectional
Chen et al., 2016 [89]	Patient group: 20. Unilaterally implanted post-lingually deaf CI users with ≥6 months experience. Age 24–77 (mean = 54.58). Controls: 20. Age 24–78 (mean = 54.89).	Visual stimuli consisting of circular checkerboard patterns in 10 s blocks. Auditory stimuli consisting of normal speech and reversed speech in 5 s blocks and tonal bursts in 3 s blocks. Loudness levels for auditory stimuli were adjusted to subjective comfortable levels.	Bilateral fNIRS. Temporal lobe ROI centered at T7/T8. Occipital lobe ROI centered at O1/O2.	Speech recognition: Oldenburg sentences test (OLSA) in quiet and noise	Cross-sectional
Chen et al., 2017 [90]	Patient group: 20. Unilaterally implanted post-lingually deaf CI users with ≥6 months experience. Age 24–77 (mean = 54.58). Controls: 20. Age 24–78 (mean = 54.89).	Visual stimuli consisting of circular checkerboard patterns in 10 s blocks. Auditory stimuli consisting of tonal bursts in 3 s blocks. Loudness levels for auditory stimuli were adjusted to subjective comfortable levels.	Bilateral fNIRS. Left and right temporal lobe and occipital lobe. Simultaneous EEG.	Speech recognition: Oldenburg sentences test (OLSA) in quiet and noise	Cross-sectional
Mushtaq et al., 2020 [78]	Patient group: 19. Bilaterally implanted CI users with 29–123 months experience. Age 6–11 (mean = 8.4). Controls: 20. Age 6–12 (mean = 9.5).	Visual speech, auditory speech, signal correlated noise, and steady speech shaped noise. On average 2.97 s long.	Bilateral fNIRS with lowermost optode close to preauricular point and uppermost optode aligned towards Cz.	Speech understanding: Bamford–Kowal–Bench (BKB) sentences in silence and in noise	Cross-sectional
Old et al., 2016 [91]	CI users: 32. Post-lingually deaf adults. Experience range 1 day–12 years. Age 23–86. Controls: 35. Adults aged 24–65	Normal speech, channelized speech, scrambled speech, environmental sounds. All at 60 dB for 20 s blocks	Bilateral fNIRS with headset centered at T7/T8. Targets lateral temporal lobe and superior temporal gyrus (LTL/STG)	Hearing level: Speech recognition threshold (SRT). Speech perception: Consonant-Nucleus-Consonant (CNC) words, AzBio Sentence Test. Both presented in quiet at 60 dB	Cross-sectional
Zhou et al., 2018 [92]	Patient group: 20. Post-lingually deaf CI users with >12 months experience with right-sided implant. Mix of unilaterally and bilaterally implanted individuals. Age 46–79 (mean = 64.2) Controls: 19. Age 33–70 (mean = 53.5).	Auditory and visual speech stimuli. 11 s long blocks. Auditory at 65 dBA.	Bilateral fNIRS. Left middle superior temporal lobe, right anterior temporal lobe, superior temporal sulcus/gyrus.	Speech understanding: Open-set consonant-nucleus-consonant (CNC) words and CUNY sentences. CNC presented in quiet at 60 dBA. CUNY presented in quiet at 60 dBA and in noise of 5–15 dB SNR.	Cross-sectional

**Table 2:** Research questions and key results from the eight included articles.

Record	Key Purpose/Questions	Summary of Main Results
Anderson et al., 2017 [86]	How does cross-modal activation of auditory brain regions by visual speech change from pre- to post-implantation? How does this relate to the ability to understand speech with a cochlear implant (CI)? What is the relationship between post-implant cortical plasticity within auditory brain regions and the ability of these regions to respond to auditory speech stimulation?	Increased cross-modal activation of auditory brain regions by lip-reading pre-implantation is not associated with post-implantation cortical responsiveness to auditory speech. Differences in pre- to post-implantation activation by visual speech is associated with speech understanding outcomes (*r* = 0.77) and with increased cross-modal activation post-implantation associated with increased auditory responsiveness and better speech understanding outcomes.
Anderson et al., 2019 [87]	To understand whether fNIRS measures of cross-modal activation obtained pre-operatively could predict future clinical outcomes for CI candidates. To explore whether pre-operative brain imaging using fNIRS could offer incremental prognostic information and value above that already provided by known clinical factors. To explore underlying mechanisms of the relationship between pre-operative brain activation and post-operative outcomes.	Stronger activation to visual speech pre-operatively was predictive of poorer speech understanding outcomes post-implantation (*r* = −0.75). fNIRS measures can provide additional prognostic information about future CI outcome. Relationship between fNIRS measurements and outcomes driven by clinical factors (i.e., whether participants were pre- or post-lingually deaf).
Chen et al., 2017 [88]	To investigate whether cross-modal functional connectivity between visual and auditory cortices is elevated in CI users. To assess the relationship between cross-modal functional connectivity and speech recognition abilities in CI users.	CI users exhibited reduced intra-modal connectivity within visual and auditory areas and greater cross-modal connectivity between visual and auditory areas in the left hemisphere. Cross-modal functional connectivity was correlated with Freiburg speech recognition scores but not OLSA scores (*r* = −0.525).
Chen et al., 2016 [89]	How does the combination of visual and auditory cortex reorganization within the same CI user jointly affect their speech recognition performance?	CI users with more reorganization of the visual cortex compared to reorganization of the auditory cortex performed better in the speech recognition tasks than CI users with the opposite pattern of reorganization (*R* = 0.518).
Chen et al., 2017 [90]	To investigate whether stimulus-specific adaptation in the visual system is enhanced in CI users compared to NH controls and whether such enhanced adaptation corresponds to decreased activity in visual cortex during visual processing.	Reduced visually evoked activation in the visual cortex and reduced auditory-evoked activation in the auditory cortex were observed in CI users compared to NH controls when fNIRS-measured latency was analyzed. CI users showed enhanced stimulus-specific adaptation for visual stimuli but decreased adaptation for auditory stimuli compared to NH controls. EEG adaptation for auditory stimuli and speech recognition scores did not correlate.
Mushtaq et al., 2020 [78]	To investigate the influence of cross-modal plasticity on speech understanding in children with CIs. To explore the relationship between speech understanding ability and intelligibility and amplitude modulation processing.	Significant activation to signal correlated noise was noted only in the CI group. Responses to visual speech were larger in the CI group than in the NH group. Responses to auditory speech were larger than responses to signal correlated noise, which were larger than responses to steady speech shaped noise. No significant correlations were noted between speech understanding scores and visual speech activation (ԏ*b* = 0.236); auditory speech activation (ԏ*b* = 0.189); intelligibility processing (ԏ*b* = −0.047); nor amplitude modulation processing (ԏ*b* = −0.142).
Old et al., 2016 [91]	To better understand speech–understanding variability in outcomes. To explore the use of fNIRS as an objective measure of speech perception.	Greater activation to speech stimuli compared to unintelligible speech in good users. Poor users showed no distinguishable differences. Ratio of activation to speech:scrambled speech was directly correlated with CNC (*R*^2^ = 0.53 to 0.68) and AzBio scores (*R*^2^ = 0.55 to 0.66). Cortical activation measures did not correlate with their general auditory sensitivity (SRT scores).
Zhou et al., 2018 [92]	To determine whether fNIRS responses to auditory or visualspeech in different brain regions correlated with speech understanding abilities in CI users.	fNIRS responses to auditory stimuli in the left middle superior temporal lobe and the right anterior temporal lobe were negatively correlated with auditory speech understanding tests scores (*r* = −0.650 and −0.620). Responses to visual stimuli in the left STS/STG were negatively correlated with auditory speech understanding scores (*r* = −0.668). Combination of the above responses produced a better prediction of auditory speech understanding ability than the activity in any one area alone (*R*^2^ = 0.709).

## Data Availability

All data pertaining to the review are contained within Table 1 and Table 2.
